# The 2003 Ed Nelson Lecture. Smoking Cessation Revisited. Is It Time to Change Our Approach?

**DOI:** 10.1186/1617-9625-1-4-233

**Published:** 2003-12-15

**Authors:** Paul McDonald

**Affiliations:** 1 Health Behaviour Research Group, University of Waterloo, Canada

## 

Clinical approaches to smoking cessation are based on a goal of increasing the odds that a given person will quit smoking. Current treatment recommendations are based on meta-analyses of randomized clinical trials conducted over short intervals. An underlying assumption is that nicotine is the primary reason it is so difficult to quit and that pharmacotherapies are the "best" way to treat the underlying dependency. Data on the relative risk of developing tobacco related disease is used as the basis for selecting priority sub-populations (e.g., those who smoke the most). But should these data be used as the foundation for creating national strategies for the treatment of smokers? The presenter will use data from Canada to offer a different view. He will argue that national strategies should not be simple extrapolations of clinical treatments or relative risk ratios. He will argue that national strategies must employ different goals and outcome measures. For example, our aim should be to use available resources to maximally reduce the health and economic burden of smoking. Policies that create supportive environments, communication campaigns that build self-awareness and efficacy, combined with a triage system that matches smokers to different types of treatment should be the foundation of our approach. Traditional clinical approaches (especially pharmacotherapy) should be used selectively rather than universally.

## About the speaker

***Paul McDonald ***is a Professor of Health Studies and Director of the Health Behaviour Research Group at the University of Waterloo. Professor McDonald is also a Principal Investigator with the Ontario Tobacco Research Unit. He holds a Ph.D. in Health Behaviour from the University of Waterloo and a M.A. in Clinical Psychology from the University of Western Ontario. Dr. McDonald has published a number of peer-reviewed papers and technical reports in the area of population based and clinical approaches to smoking cessation. His current research aims to improve the health impact and cost efficiency of smoking cessation treatments, communication campaigns and policies.

Dr. McDonald works closely with program providers and decision makers in government and non-profit agencies to develop and test tobacco control interventions. He is a principal designer of Canada's largest telephone helpline for smokers, and the author of some of the most widely used self-help smoking cessation booklets and websites in Canada. He has recently completed a consultation paper for the Government of Canada that recommended a national framework for smoking cessation.

We are delighted to have Paul McDonald agree to present the inaugural Ed Nelson Lecture. This presentation is named in honour of the Society's founder, who sadly passed away in the summer of 2002.

## Competing interests

The authors declare that they have no competing interests.

